# Empowering Modern Dentistry: The Impact of Artificial Intelligence on Patient Care and Clinical Decision Making

**DOI:** 10.3390/diagnostics14121260

**Published:** 2024-06-14

**Authors:** Zeliha Merve Semerci, Selmi Yardımcı

**Affiliations:** Department of Oral and Maxillofacial Radiology, Faculty of Dentistry, Akdeniz University, Antalya 07070, Turkey

**Keywords:** artificial intelligence, dentistry, convolutional neural networks, patient care, diagnostic accuracy

## Abstract

Advancements in artificial intelligence (AI) are poised to catalyze a transformative shift across diverse dental disciplines including endodontics, oral radiology, orthodontics, pediatric dentistry, periodontology, prosthodontics, and restorative dentistry. This narrative review delineates the burgeoning role of AI in enhancing diagnostic precision, streamlining treatment planning, and potentially unveiling innovative therapeutic modalities, thereby elevating patient care standards. Recent analyses corroborate the superiority of AI-assisted methodologies over conventional techniques, affirming their capacity for personalization, accuracy, and efficiency in dental care. Central to these AI applications are convolutional neural networks and deep learning models, which have demonstrated efficacy in diagnosis, prognosis, and therapeutic decision making, in some instances surpassing traditional methods in complex cases. Despite these advancements, the integration of AI into clinical practice is accompanied by challenges, such as data security concerns, the demand for transparency in AI-generated outcomes, and the imperative for ongoing validation to establish the reliability and applicability of AI tools. This review underscores the prospective benefits of AI in dental practice, envisioning AI not as a replacement for dental professionals but as an adjunctive tool that fortifies the dental profession. While AI heralds improvements in diagnostics, treatment planning, and personalized care, ethical and practical considerations must be meticulously navigated to ensure responsible development of AI in dentistry.

## 1. Introduction

Artificial intelligence (AI) stands as a transformative power in today’s digital revolution, affecting various economic sectors by performing tasks that typically require human intelligence. Its introduction to dentistry is particularly notable, offering new and improved ways to enhance diagnostic imaging, plan treatments, and manage patient care. This review aims to examine how AI is developing within dentistry and its significant influence on the dental profession.

Starting with the basics and moving to the latest advances, AI techniques like deep learning and neural networks are now used for identifying dental conditions, making treatment decisions, planning treatments, predicting outcomes, and forecasting the progression of diseases. These AI approaches are proving to be highly capable, in certain complex cases outperforming humans through the analysis of diverse data. This is especially apparent in AI’s role in improving the accuracy of dental imaging and aiding in clinical decisions [1].

AI encompasses a broad range of technologies designed to simulate human intelligence, including natural language processing, computer vision, and machine learning. In dentistry, AI applications primarily involve machine learning (ML) and deep learning (DL) algorithms, which are capable of analyzing large datasets, recognizing patterns, and making predictions based on learned information. Key AI technologies used in dental applications include the following: supervised learning, unsupervised learning, reinforcement learning, and deep learning. Supervised learning approach involves training AI models on labeled datasets, enabling them to predict outcomes based on new, unseen data. Supervised learning is widely used in dental imaging for tasks such as detecting caries, identifying root fractures, and classifying dental restorations. Unlike supervised learning, unsupervised learning algorithms identify patterns in unlabeled data. While less common in dentistry, unsupervised learning can be valuable for discovering new insights from large datasets without predefined labels. Reinforcement learning involves training AI models to make decisions by rewarding desired outcomes. Although still emerging in dental applications, reinforcement learning holds potential for optimizing treatment plans and adaptive learning systems. A subset of ML, deep learning involves neural networks with multiple layers (deep neural networks). CNNs, a type of deep learning model, are particularly effective in image analysis and have been extensively applied in dental radiology and pathology.

In delving into the fusion of AI with various dental disciplines, this review illuminates its integration in applications such as dentition restoration detection, caries lesion identification, and periapical infection diagnosis, highlighting a promising horizon for AI’s role in dentistry [2]. Concurrently, the synergy between evidence-based dentistry (EBD) and intelligent technology is scrutinized. This necessitates ongoing research to surmount developmental challenges and fully harness AI’s potential. By embracing both the innovations and the intricate challenges, this review endeavors to articulate a comprehensive perspective on the future of AI in dental medicine.

The motivation behind this research stems from the growing body of evidence suggesting that AI can outperform conventional diagnostic methods in several dental applications. AI algorithms, particularly those involving convolutional neural networks (CNNs) and deep learning frameworks, have demonstrated superior accuracy and efficiency in diagnosing dental conditions, planning treatments, and predicting outcomes. Despite these promising advancements, the integration of AI into routine dental practice faces numerous challenges, including methodological limitations, regulatory hurdles, and ethical considerations.

This review aims to provide a comprehensive overview of the current state of AI in dentistry, highlighting both the advancements and challenges associated with its application. By synthesizing findings from recent studies, this review seeks to elucidate the potential of AI to transform dental practice, identify the gaps and limitations in current methodologies, and propose directions for future research and clinical implementation.

### Literature Search and Selection

In conducting this narrative review on the impact of artificial intelligence (AI) on patient care and clinical decision making in dentistry, a comprehensive literature search was performed to identify relevant studies and articles. The search strategy focused on three primary databases: PubMed, Scopus, and Google Scholar. The keywords used in the search included “artificial intelligence”, “dentistry”, “convolutional neural networks”, “deep learning”, “diagnostic accuracy”, and “clinical decision-making”.

The initial search yielded a substantial number of articles, which were then screened based on their titles and abstracts.

Inclusion criteria for the articles included the following:

- Publications that discussed the role of artificial intelligence in various dental disciplines such as endodontics, oral radiology, orthodontics, pediatric dentistry, periodontology, prosthodontics, and restorative dentistry.
- Studies that presented both quantitative and qualitative data on the effectiveness of AI in dental diagnostics and treatment.
- Articles published in English within the last two decades, given the rapid advancements in AI and its applications in medical imaging and diagnostics.
- Exclusion criteria were as follows:
- Studies that focused solely on non-dental applications of AI.
- Articles that did not provide substantial information on the use of AI in dental practice.
- Publications in languages other than English.

After the initial screening, full-text reviews of the selected articles were conducted to ensure their relevance and to extract detailed information about the methodologies, findings, and implications of AI applications in dentistry. The final selection included studies that highlighted significant advancements in AI technology, its integration into clinical practice, and its impact on improving diagnostic accuracy and patient outcomes.

## 2. AI in Endodontics

Recent studies harness sophisticated neural network frameworks to advance dental diagnostic capabilities through enhanced imaging techniques in endodontics (Table 1). Research conducted by Kirnbauer et al. and Pauwels et al. integrated Spatial Configuration-Net (SCN) and convolutional neural networks (CNNs) to achieve high precision in detecting periapical lesions and accurately locating teeth within cone beam computed tomography (CBCT) images [3,4]. Similarly, Altındag et al. employed the Mask R-CNN model to detect pulp stones in dental radiographs with notable accuracy and sensitivity [5]. Furthering this body of work, Johari et al. explored the application of probabilistic neural networks to identify vertical root fractures, yielding encouraging results in diagnostic efficacy [6]. Comparative analyses, such as those by Hiraiwa et al. and Hu et al., have assessed AI’s diagnostic competencies against human radiologists, frequently demonstrating AI’s enhanced performance in tasks such as appraising root morphologies and diagnosing vertical root fractures [7,8]. However, these studies have also brought to light several challenges, including issues with dataset balance, image noise, and the need for larger and more varied datasets to refine the AI models [9,10].

## 3. AI in Oral Radiology

Recent studies have demonstrated the potential of AI and machine learning (ML) in revolutionizing dental diagnostics and treatment planning through advanced imaging technologies and algorithms (Table 2). Yılmaz et al. developed a computer-assisted detection system using CBCT images to classify periapical cysts and keratocystic odontogenic tumors, with an impressive 94% accuracy using SVM classifiers [11]. Kuwada et al. evaluated deep learning models for detecting maxillary impacted supernumerary teeth in panoramic radiographs, with DetectNet showing superior diagnostic efficacy [12]. Lee et al. utilized a pre-trained GoogLeNet Inception v3 for diagnosing dental caries from periapical radiographs, achieving high diagnostic performance with accuracy rates of up to 89.0% [13]. Cantu et al. showed a deep learning neural network outperformed experienced dentists in detecting caries lesions on bitewing radiographs, indicating significant potential for AI in improving diagnostic standards [14]. Shaheen et al. developed a deep learning-based AI framework for tooth segmentation on CBCT images, drastically reducing clinical workloads and enhancing diagnostic efficiency [15]. Ariji et al. created a model to detect and classify mandibular radiolucent lesions with high sensitivity, especially for dentigerous cysts [16]. Fukuda et al. explored the use of convolutional neural networks in classifying the relationship between the mandibular third molar and the canal, achieving diagnostic performance comparable to experienced radiologists [17]. Tajima et al. developed an AI system for detecting cyst-like radiolucent lesions in the jaw with high accuracy, sensitivity, and specificity, underscoring the potential of AI in general dental clinics for lesion detection [18]. Baydar et al. developed an AI model to evaluate bite-wing radiographs with high precision, suggesting its potential to improve diagnostic accuracy in dental practices [19]. Mackie et al. explored machine learning and radiomics in diagnosing temporomandibular joint osteoarthritis using high-resolution CBCT scans, highlighting the significance of integrating machine learning with detailed imaging and biological data [20]. Orhan et al. tested a CNN-based AI system for detecting periapical pathosis in CBCT images with 92.8% accuracy, emphasizing the effectiveness of CNNs in dental radiographic analysis [21]. Kuwana et al. explored the effectiveness of deep learning in identifying lesions in the maxillary sinuses from panoramic radiographs, achieving a 100% detection rate for healthy sinuses [22]. Minnema et al. compared a novel mixed-scale dense CNN architecture against traditional models for metal artifact reduction in CBCT scans, showing significant improvements in accuracy [23]. Lee et al. used a deep CNN to diagnose odontogenic cystic lesions from dental images, significantly outperforming models using panoramic images [24]. Basaran et al. developed an AI model to diagnose dental conditions using panoramic radiography, showing promise in detecting various dental conditions, albeit with limitations in caries and dental calculus detection [25]. Mima et al. introduced a method utilizing Faster R-CNNs for detecting and classifying tooth regions in dental panoramic X-ray images with high precision and accuracy [26]. Zhu et al. introduced an AI framework for diagnosing common dental diseases from panoramic radiographs, showing high specificity for all diseases except for caries, where it showed lower sensitivity and specificity, but overall, it demonstrated superior diagnostic performance compared to dentists in certain areas [27].

## 4. AI in Oral Surgery

Recent advancements in oral research have highlighted the significant impact of AI and deep learning technologies in improving diagnostic accuracy, treatment planning, and clinical outcomes in oral surgery (Table 3). Lee et al. evaluated an automated deep convolutional neural network (DCNN) for classifying dental implant systems from radiographic images, demonstrating high accuracy and surpassing most dental professionals [28]. Matsuda et al. analyzed the anatomical aspects of horizontally impacted mandibular third molars in young Japanese patients, identifying predictors for lingual cortical bone perforation [29]. The study provides valuable insights for treatments involving impacted mandibular third molars, underscoring the importance of considering specific factors such as gender, age, and available space. A recent study also investigated the use of ResNet models for diagnosing the need for orthognathic surgery based on cephalometric radiograph images. Their findings suggested that an AI model structure significantly impacts medical image predictions. This research is crucial for enhancing orthognathic surgery planning and highlights the effectiveness of AI in cephalometric analysis. Zhang et al. employed artificial neural networks to predict facial swelling after impacted mandibular third molar extraction with high accuracy. Their study demonstrates the potential of AI in predicting post-surgical outcomes, facilitating better patient care and clinical decision making [30,31]. Tanikawa et al. explored AI’s capability to predict 3-D facial morphology changes following orthognathic surgery and orthodontic treatment. Their systems demonstrated high accuracy in anticipating post-treatment facial topography, indicating the potential of AI in planning various facial treatments, including aging, cancer surgery, or cosmetic interventions [32]. Choi et al. developed an AI model using a deep convolutional neural network to determine the spatial relationship between the mandibular third molar and the inferior alveolar nerve from panoramic radiographs. This model showed superior diagnostic accuracy compared to experienced oral and maxillofacial surgeons, suggesting AI could significantly aid surgical planning and reduce the need for CBCT scans [33].

## 5. AI in Orthodontics

Artificial intelligence can be utilized for different purposes in orthodontics, including diagnostic treatment analysis, the identification of anatomical landmarks, orthodontic treatment with or without extractions, skeletal classification, determination of growth and development phases, and orthognathic surgical planning (Table 4). Niño-Sandoval et al. explored mandibular morphology using artificial neural networks (ANNs) on lateral radiographs, demonstrating improved prediction accuracy for mandibular dimensions. According to their findings, for facial reconstruction and victim identification, ANNs can be utilized in orthodontic treatments and craniofacial studies [35]. Recent studies have focused on cephalometric landmark identification using AI, with convolutional neural networks (CNNs) and fully automated AI-assisted analysis. According to Panesar et al., the precision and accuracy of the examiners with the aid of AI improved by 10.47% and 27.27%, respectively [36,37]. Kochhar et al. assessed bone volume in CBCT scans of patients with a unilateral cleft lip and/or palate using OsiriX software. They found that OsiriX software provided good reliability in measurements of bone volume for evaluating alveolar cleft defects [38]. Çoban et al. evaluated the reliability of digital manual versus AI-based automatic cephalometric analyses, finding significant discrepancies in measurements across different sagittal skeletal malocclusions. The study highlights AI’s potential for preliminary orthodontic assessment and the need for further validation across malocclusion groups [39]. Jiang introduced “CephNet”, a highly accurate AI system for automating cephalometric analysis, achieving a classification accuracy of 89.33% [40]. Li et al. developed artificial neural networks to predict orthodontic treatment plans including the determination of extraction/non-extraction, extraction patterns, and anchorage patterns. Their research suggests ANNs as a valuable tool for assisting less experienced orthodontists in treatment planning [41]. Silva et al. assessed CEFBOT, an AI-based cephalometric software, comparing its performance to a trained human examiner. Despite some limitations, CEFBOT demonstrated high reliability in completing cephalometric measurements, indicating its potential as a tool to enhance radiologists’ skill set [42].

## 6. AI in Pediatric Dentistry

Recent studies across various domains of pediatric dentistry have utilized advanced machine learning and AI techniques to improve diagnostic accuracy, predict dental conditions, and explore new biomarkers for early childhood caries (ECC) (Table 5). Ramos-Gómez et al. employed a random forest algorithm to predict active dental caries in children based on a parental oral health questionnaire. Key predictors included parent age, unmet dental needs, and the child’s oral health status, demonstrating the machine learning approach’s efficacy in caries risk prediction [43]. Zhao et al. developed a deep learning model, HeadNet, for a non-invasive adenoid hypertrophy assessment using lateral cephalograms. The model’s system achieved high sensitivity (0.906), specificity (0.938), and accuracy (0.919) for adenoid hypertrophy assessment and offers an efficient alternative to traditional invasive methods, potentially leading to earlier diagnoses in pediatric patients [44]. Ahn et al. utilized deep convolutional neural networks to detect mesiodens in panoramic radiographs of children with mixed dentition. Their findings highlighted the potential of AI models for a more accurate and faster diagnosis, particularly ResNet-101 and Inception-ResNet-V2, with accuracy, precision, recall and F1 scores higher than 90% [45]. Çalışkan et al. explored the effectiveness of a deep learning-based convolutional neural network in detecting and classifying submerged molars in panoramic radiographs. The AI system demonstrated superior accuracy compared to dental experts, indicating its potential to enhance diagnostic reliability in pediatric dentistry [46]. Bilgir et al. utilized a R-CNN Inception v2 model to develop an AI system, (CranioCatch, Eskisehir, Turkey) for detecting and numbering teeth on panoramic radiographs. The system showed high sensitivity and precision (0.955 and 0.965, respectively), underlining AI’s underutilized potential in dentistry for automated dental charting and diagnosis [47]. You et al. presented a deep learning-based AI model for detecting dental plaque on primary teeth. The AI model presented clinically acceptable performance in detecting dental plaque on primary teeth compared to specialists [48]. Koopaie et al. investigated salivary cystatin S levels as a biomarker for ECC, employing machine learning methods to analyze saliva samples. The findings indicated that incorporating measurements of cystatin S in saliva improved the performance of machine learning techniques in distinguishing between children with early tooth decay and those without any cavities [49].

## 7. AI in Periodontology

Numerous studies have been conducted in periodontology regarding the applications of AI and deep CNNs. The detection of alveolar bone loss and early changes in bone density can be identified using AI models (Table 6). Furthermore, it is considered that deep learning methods could be utilized in situations where early intervention may be necessary regarding implants and surrounding tissues. Chau et al. developed an AI system to detect gingivitis from intraoral photographs, achieving high accuracy and demonstrating the potential of AI in identifying gingival inflammation [51]. Lin et al. introduced an automatic system for measuring alveolar bone loss in periodontitis patients using periapical radiographs. They concluded that the proposed automatic system can effectively estimate the grade of horizontal alveolar bone loss in periodontitis radiograph images [52]. Chang et al. explored combining deep learning with computer-aided design (CAD) processing to diagnose and stage periodontitis from dental panoramic radiographs. The approach achieved high accuracy in detecting periodontal bone levels and classifying periodontal bone loss, showcasing the effectiveness of deep learning hybrid frameworks in automating the classification process of periodontitis [53]. Thanathornwong et al. employed a deep learning-based faster regional convolutional neural network (faster R-CNN) to detect periodontally compromised teeth in digital panoramic radiographs. The model achieved a sensitivity of 0.84, a specificity of 0.88, and an F-measure of 0.81 [54]. Lee et al. developed a computer-assisted detection system using deep CNNs for diagnosing and predicting periodontally compromised teeth (PCT) with promising accuracy. With the proposed deep learning algorithm, the diagnostic accuracy for PCT was 81.0% for premolars and 76.7% for molars and the accuracy of predicting extraction was 82.8% for premolars and 73.4% for molars [55].

## 8. AI in Prosthodontics

The adoption of AI technology in all prosthetic procedures is still met with some hesitation. AI systems are particularly useful for classifying outcomes and processing and analyzing repetitive workflows. AI algorithms can also support decision making for less experienced practitioners and facilitate case analysis (Table 7). Despite prevailing misconceptions and some limitations related to AI, it continues to evolve due to its advantages in providing accurate diagnoses. The applications of AI in prosthetic treatment include prosthetic planning and design, CAD/CAM applications, digital smile design, color selection, temporomandibular joint disorders, and occlusion. Lee et al. demonstrated the use of deep CNN algorithms, specifically GoogLeNet Inception v3, in identifying and classifying dental implant systems from radiographs with high accuracy [57]. Bayrakdar et al. evaluated an AI system’s ability to analyze bone height and thickness for dental implant planning using CBCT images. The system indicated that the accuracy of correct detection was 72.2% for canals, 66.4% for sinuses/fossae, and 95.3% for regions with missing teeth, demonstrating the potential of deep learning to enhance diagnostic precision and treatment planning in dentistry, especially in the field of implantology [58]. Lerner et al. explored the creation of implant-supported monolithic zirconia crowns through a fully digital procedure facilitated by AI. The study reported a high success rate in prosthetic rehabilitation, highlighting AI’s narrow application in dental CAD processes but suggesting the need for broader research and validation [59]. Takahashi et al. utilized the Yolov3 object detection algorithm to identify dental implant systems in panoramic radiographs. Despite achieving good accuracy, especially for specific implants, the study noted the requirement for more diverse implant system images to enhance clinical application [60]. Yamaguchi et al. employed a CNN to predict the debonding of CAD/CAM composite resin crowns from 2D images of 3D models, achieving high accuracy, precision, recall, and F-measure values (98.5%, 97.0%, 100%, and 0.985, respectively) [61].

## 9. AI in Restorative Dentistry

Recently, increasing aesthetic expectations have significantly elevated the importance of diagnosis and treatment processes within the field of restorative dentistry. With the advancement of technology, the evaluation of artificial intelligence applications has commenced. In restorative dentistry, artificial intelligence applications can be enumerated as caries detection, dental plaque identification, the differentiation of developmental lesions, robotic cavity preparation, color matching, the assessment of restoration success, and material discrimination (Table 8).

Aslan et al. developed an AI algorithm for detecting and classifying dental restorations from panoramic radiographs with high accuracy, illustrating AI’s potential to improve radiographic reports and dentist–patient communication. The algorithm was able to identify various restorations with an overall precision of 93.6% [62]. Schwendicke et al. utilized CNNs to detect caries lesions in near-infrared light transillumination (NILT) images, aiming to refine caries detection and potentially expand dental examinations to non-traditional settings. Despite moderate accuracy, the combination of NILT and CNNs promises improvements in caries diagnosis comparable to traditional radiographic methods [63]. Fontenele et al. investigated an AI-driven tool for automatic tooth segmentation in cone beam computed tomography images, highlighting how dental fillings affect performance. The study demonstrated the tool’s high accuracy and speed, emphasizing its clinical acceptability and potential to enhance digital dental workflows [64]. Zheng et al. compared three CNNs in analyzing the radiographic penetration depth of carious lesions, with ResNet18 showing superior accuracy. Integrating clinical parameters with ResNet18 further improved its diagnostic performance, suggesting the effectiveness of a multi-modal CNN approach in automating the diagnosis of deep caries and pulpitis [65]. Güneç et al. compared the efficacy of an AI application against junior dentists in diagnosing dental caries and periapical infections. The AI system, which was trained using comprehensive datasets of panoramic dental X-rays, surpassed the performance of junior dentists by delivering results that were both more precise and quicker [66].

**Table 8:** Advanced AI applications in restorative dentistry.

Authors	Summarized Abstract	Methods Used	Results	Conclusions
Zheng [65]	- Diagnosis of deep caries and pulpitis using convolutional neural networks.	- Assessed each CNN for accuracy, precision, sensitivity, specificity, and AUC. - Employed Grad-CAM to identify critical image features influencing CNN decisions.	- Integrated clinical parameters to enhance CNN performance. - Utilized Grad-CAM to identify important image features for CNNs.	- Multimodal CNN enhanced performance when integrated with clinical parameters.
Fontenele [64]	- Study evaluated AI tool performance for tooth segmentation on CBCT images.	- AI convolutional neural networks assessed segmentation performance for control and experimental groups.	- Dental fillings significantly influenced segmentation performance, showing high accuracy metrics. - AI-driven tool provided 3D tooth models from CBCT images efficiently.	- Dental fillings significantly influenced segmentation performance, but AI tool showed accuracy.
Schwendicke [63]	- Deep learning used for caries lesion detection in near-infrared light images.	- Resnet18 and Resnext50 were trained with data augmentation and 10-fold cross-validation, applying a one-cycle learning rate policy.	- Resnext50 model had a mean AUC of 0.74. - Model was sensitive to areas affected by caries lesions.	- Moderately deep CNN showed satisfying ability to detect caries lesions. - CNNs may assist in NILT-based caries detection in various dental settings.
Abdalla-Aslan [62]	- AI system detected and classified dental restorations on panoramic radiographs.	- Features related to shape and gray-level distribution were extracted and used to classify restorations into 11 categories via a trained algorithm.	- Algorithm detected 94.6% of restorations, with 93.6% classification accuracy.	- Machine learning algorithm excelled in detecting and classifying dental restorations.

## 10. Discussion

The integration of artificial intelligence in various dental disciplines, as presented in this review, underscores its transformative potential to enhance diagnostic accuracy, treatment planning, and overall patient care. The AI methodologies and applications discussed across endodontics, oral radiology, orthodontics, pediatric dentistry, periodontology, prosthodontics, and restorative dentistry collectively demonstrate significant advancements.

In endodontics, AI has shown remarkable potential in enhancing diagnostic capabilities, particularly in identifying vertical root fractures and root morphology. Studies by Hu et al. and Fukuda et al. demonstrate the efficacy of deep learning models like ResNet50 and CNNs in diagnosing VRFs from CBCT and panoramic radiography images, often surpassing human radiologists in accuracy and sensitivity [7,9]. The integration of AI in endodontics can lead to more precise and timely diagnoses, reducing the likelihood of missed fractures and improving treatment outcomes. However, the need for large annotated datasets and the potential for model overfitting remain challenges that must be addressed. Oral radiology has significantly benefited from AI applications, particularly in the detection and classification of dental diseases from radiographic images. The studies by Zhu et al. and Mima et al. illustrate the use of AI frameworks based on BDU-Net and faster R-CNNs for diagnosing dental diseases and detecting individual teeth with high specificity and efficiency [26,27]. These advancements can streamline the diagnostic process, reduce diagnostic errors, and enable more personalized treatment plans. The main implication is the potential for AI to serve as an adjunctive tool, enhancing the capabilities of radiologists and improving patient outcomes. AI applications in orthodontics primarily focus on cephalometric analysis and treatment planning. The work by Panesar et al. and Alessandri-Bonetti et al. shows that AI-assisted cephalometric analysis can significantly improve precision and accuracy [36,37]. AI can automate the labor-intensive process of landmark identification, making it faster and more reliable. This can free up valuable time for orthodontists, allowing them to focus more on patient care. In pediatric dentistry, AI has been employed to detect dental plaque, identify taurodontism, and classify mesiodens with high accuracy. Studies by You et al. and Duman et al. highlight the effectiveness of AI models in these applications, demonstrating performance comparable to expert clinicians [48,50]. The use of AI can improve early detection and intervention, which is particularly important in pediatric patients to prevent long-term dental issues. The implication here is the potential for AI to enhance preventive care and early diagnosis, ultimately improving oral health outcomes in children. Periodontology has seen the development of AI systems for detecting gingivitis, measuring alveolar bone loss, and diagnosing periodontitis. Research by Chau et al. and Lin et al. showcases AI’s ability to accurately diagnose periodontal conditions from intraoral photographs and radiographs [51,52]. These tools can aid in the early detection and monitoring of periodontal diseases, leading to more effective and timely interventions. In prosthodontics, AI applications include the identification and classification of dental implant systems, as well as the prediction of crown debonding probability. Studies by Lee et al. and Yamaguchi et al. demonstrate the high accuracy of AI models in these tasks, which can improve implant planning and maintenance [57,61]. AI’s ability to predict potential issues such as debonding can help in preventive maintenance and extend the lifespan of prosthetic devices. The implication is a potential reduction in prosthetic failures and enhanced patient satisfaction, though the initial setup and training of these AI systems may require significant investment. Restorative dentistry benefits from AI through enhanced caries detection, tooth segmentation, and restoration classification. Research by Zheng et al. and Abdalla-Aslan et al. highlights AI’s capability to detect caries and classify dental restorations with high precision, outperforming traditional methods. This can lead to more accurate diagnoses and effective treatment plans, ultimately improving patient outcomes [62,65]. The challenge remains in ensuring the AI models are robust and can generalize well across diverse patient populations and varying clinical scenarios.

Despite the promising advancements of AI in dental practice, several limitations need to be addressed. Different methodological approaches in machine learning, such as supervised learning, unsupervised learning, and reinforcement learning, each have inherent strengths and weaknesses that impact their application in dentistry. For instance, supervised learning requires large, labeled datasets, which are often difficult to obtain in medical fields due to privacy concerns and the need for expert annotation. Unsupervised learning, while useful for discovering hidden patterns in data, may not always provide clinically actionable insights without additional validation. Moreover, the choice of AI models, such as convolutional neural networks for image analysis or recurrent neural networks for time-series data, significantly influences the outcomes. Each model type requires specific tuning and validation to ensure accuracy and reliability in different dental applications. Additionally, there is a lack of standardization in the methodologies used across studies, making it difficult to compare results and draw definitive conclusions about the best practices for AI in dentistry [67,68].

Regulatory approval is another critical factor that affects the deployment of AI in dental practice. AI systems must undergo rigorous testing and validation to meet the standards set by regulatory bodies such as the U.S. Food and Drug Administration (FDA), the European Medicines Agency (EMA), and other national health authorities. These approvals ensure that AI tools are safe, effective, and reliable for clinical use [69,70]. However, the regulatory pathways for AI are still evolving, and navigating these requirements can be complex and time consuming. This regulatory landscape creates a barrier to the rapid adoption of AI technologies in clinical settings [71].

Several difficulties hinder the widespread application of AI in dental practice. These include the following: data privacy and security, interoperability, cost and accessibility, training, and expertise. Ensuring the confidentiality and security of patient data used to train AI models is paramount. The use of large datasets in AI development raises concerns about data breaches and the misuse of sensitive information. Integrating AI systems with existing dental practice management software and electronic health records requires seamless interoperability, which is often challenging due to varied data formats and standards. The high costs associated with developing and implementing AI technologies can be prohibitive for many dental practices, particularly smaller ones or those in resource-limited settings. Dental professionals need adequate training to effectively use AI tools. There is also a need for interdisciplinary collaboration between dentists, data scientists, and engineers to develop and refine AI applications [72,73,74].

The development and use of AI in dentistry must adhere to established standards and guidelines to ensure consistency, accuracy, and safety. Organizations such as the American Dental Association (ADA) and the International Organization for Standardization (ISO) are working towards creating guidelines for the ethical and effective use of AI in dental practice. These standards encompass aspects such as data quality, model validation, and clinical integration, providing a framework for the responsible development and deployment of AI technologies. The ethical and legal challenges associated with AI in dentistry are significant and multifaceted. Determining who is responsible for errors or adverse outcomes associated with AI-generated decisions is a major concern. Legal frameworks need to address liability and accountability in the use of AI tools. Ensuring transparency in AI algorithms is crucial for building trust among dental professionals and patients. Black-box AI models, whose decision-making processes are not easily interpretable, pose challenges for clinical acceptance and regulatory approval. AI systems must be designed and tested to avoid biases that could lead to disparities in dental care. This includes ensuring diverse and representative datasets during the training phase to prevent skewed outcomes that favor certain populations over others [72,73].

This narrative review comprehensively covers AI applications across multiple dental disciplines, offering detailed methodological analysis and identifying future research directions. It emphasizes ethical considerations and interdisciplinary collaboration. However, the review relies on the existing literature, which may have biases and limitations, and the rapidly evolving nature of AI means some findings may become outdated. Further research should focus on conducting systematic reviews and meta-analyses to consolidate findings, as well as exploring practical implementation strategies and addressing gaps in data diversity and standardization.

## 11. Conclusions

The use of AI is both a significant change in itself and has the aim to improve patients’ care by using a new technology that will improve diagnostic precision as well as change the workflow and the approach of treatment planning. This overview, however, has made us take a glance at the types of dentistry specialties that have been aided by AI such as endodontics, oral radiology, orthodontics, pediatric dentistry, periodontology, prosthodontics, and restorative dentistry. Recent studies clearly demonstrate that AI in dentistry is poised to significantly enhance the future by offering comprehensive conveniences. Despite the numerous benefits mentioned above, the absence of extensive studies on legal issues, such as who will be held accountable for errors associated with AI or who will verify the diagnoses, indicates that AI algorithms will remain as tools to assist clinicians rather than replace them. AI research already occupies a significant position in the literature. These algorithms aim to reduce errors in diagnosis and treatment planning for clinicians burdened with heavy workloads and to minimize human-induced mistakes. The systems are expected to provide significant benefits in terms of patient health, cost, and time, especially in healthcare centers facing clinician shortages. AI’s capacity for analyzing raw data also provides the grounds for a new area of research and advances in treatment with potential for technology and scientific outbursts. Another characteristic of intelligence that calls for professionals having improvement learning is that intelligence is changing all the time; therefore, professionals need to aspire to be always motivated, informed, and in line with technology. In conclusion, AI is expected to change dentistry in a meaningful way, from current abilities, future precision, and most importantly, patients care, given that the place of AI in medicine is still the subject of discussion and patients should be included in the deliberation.

## Figures and Tables

**Table 1:** Advanced AI applications in endodontics.

Authors	Summarized Abstract	Methods Used	Results	Conclusions
Hu Z. [7]	- Deep learning models diagnose vertical root fractures (VRF) in vivo on CBCT images.	- Deep learning networks used: ResNet50, VGG19, and DenseNet169, with a training to testing ratio of 3:1.	- ResNet50 showed higher diagnostic efficiency than VGG19, DenseNet169, and radiologist.	- Deep learning models are promising for screening VRF teeth.
Fukuda M. [9]	- CNN system detects vertical root fractures on panoramic radiography images.	- A CNN-based deep learning model developed using DetectNet within DIGITS version 5.0 software.	- In total, 267 out of 330 VRFs were detected in the study. - Recall was 0.75, precision 0.93, and F measure 0.83.	- CNN model shows promise in detecting vertical root fractures on radiography.
Johari M. [6]	- CNN model detects VRF in teeth using radiographs.	- Probabilistic neural network (PNN) trained to diagnose and classify teeth with and without VRFs.	- CBCT images showed higher accuracy, sensitivity, and specificity than periapical radiographs.	- Neural network effective in diagnosing VRFs on CBCT images. - CBCT images more effective than periapical radiographs for VRF diagnosis.
Hiraiwa T. [8]	- Deep learning system accurately classifies root morphology on panoramic radiographs.	- Data augmentation process enhanced training image patches for deep learning. AlexNet and GoogleNet were utilized.	- In total, 21.4% of distal roots had extra roots on CBCT images. - Deep learning system had 86.9% accuracy in root morphology classification.	- Deep learning system accurately diagnose single or extra roots. - High accuracy in differential diagnosis of distal roots in molars.
Altındag A. [5]	- Study on deep learning model for pulp stone detection.	- Pulp stones were marked using the CranioCatch (CranioCatch, Eskişehir, Turkey) labeling program. - Mask R-CNN architecture was utilized for the deep learning model.	- Deep learning model achieved 90% sensitivity in detecting pulp stones.	- Deep learning detects pulp stones, aiding clinicians in diagnosis. - Larger datasets enhance accuracy of deep learning systems.
Pauwels R. [3]	- A comparison study between convolutional neural networks and human observers for detection of periapical lesions on intraoral radiographs.	- CNN performance on validation data compared with three oral radiologists in terms of sensitivity, specificity, and ROC-AUC.	- Mean sensitivity, specificity, and ROC-AUC values for CNN were 0.79, 0.88, and 0.86. - Radiologists had values of 0.58, 0.83, and 0.75, respectively.	- CNNs show promise in periapical lesion detection with high accuracy.
Kirnbauer B. [4]	- Deep CNN for automated detection of osteolytic periapical lesions in CBCT data.	- Two-step approach for automatic detection of periapical lesions: Spatial Configuration-Net for tooth localization, modified U-Net architecture for segmentation of lesions.	- Tooth localization network success rate: 72.6% to 97.3%. - Lesion detection sensitivity: 97.1%, specificity: 88.0%.	- Automated method shows excellent results in detecting osteolytic periapical lesions.
Gao X. [10]	- Study evaluates neural network for predicting postoperative pain after root canal.	- Back propagation (BP) neural network model developed using MATLAB 7.0.	- BP neural network model accuracy was 95.60% for pain prediction. - ANN used to predict postoperative pain with high accuracy.	- ANN model can be used to predict postoperative pain effectively.

**Table 2:** Advanced AI applications in oral radiology.

Author	Summarized Abstract	Methods Used	Results	Conclusions
Zhu J. [27]	- AI framework for dental disease diagnosis on panoramic radiographs.	- Developed AI framework based on BDU-Net and nnU-Net. - Dentists with different levels of seniority independently diagnosed the evaluation dataset.	- AI framework showed high specificity in diagnosing dental diseases. - Performance comparable to dentists with 3–10 years of experience.	- Diagnostic time of the AI framework was significantly shorter.
Mima Y. [26]	- Tooth detection method using Faster R-CNN for dental X-ray images.	- Applied Faster R-CNN to extract a rectangular area including all teeth from each X-ray image. - Classified bounding boxes for each tooth into one of 32 tooth types using the trained Faster R-CNNs.	- Detection rate per tooth: 98.9%. - Mean intersection over union for each tooth: 0.748.	- Improved tooth detection with Faster R-CNNs in divided areas.
Başaran [25]	- Developed AI model for diagnostic charting in panoramic radiography.	- Developed an AI model (CranioCatch, Eskişehir, Turkey) using a deep CNN method. Employed the Faster R-CNN Inception v2 architecture from the TensorFlow library for model implementation.	- Highest precision for prosthesis, implant supported prosthesis, lowest for caries, dental calculus.	- AI model detected dental conditions in panoramic radiographs, aiding diagnosis and treatment. - Highest sensitivity for prosthesis, implant, impacted tooth, lowest for caries, dental calculus.
Lee [24]	- Deep learning neural network for cystic lesion diagnosis in imaging.	- Periapical radiographic images split into training/validation. - Utilized a pre-trained GoogLeNet Inception v3 CNN for preprocessing and transfer learning.	- Deep CNN achieved 84.6% accuracy with panoramic images, 91.4% with CBCT.	- Deep CNN architecture enhanced diagnosis of cystic lesions. - Further studies need to include ameloblastoma in the dataset.
Minnema [23]	- MS-D network developed for bone segmentation in CBCT scans.	- Bone segmentation performance was evaluated using a leave-2-out cross-validation method. - MS-D network’s performance was compared against a clinical snake evolution algorithm and two CNN architectures.	- MS-D network accurately segmented bony structures. ResNet introduced wave-like artifacts, U-Net mislabeled background voxels.	- The MS-D network can be utilized for segmentation of bony structures in CBCT scans.
Kuwana [22]	- AI system detects periapical pathosis on CBCT images accurately.	- Learning process conducted over 1000 epochs using DetectNet with training images and labels to create a learning model.	- Reliability of correctly detecting a periapical lesion was 92.8%. - Volume measurements by AI and humans were comparable.	- AI systems accurately detected periapical lesions with high reliability. - Deep learning AI useful for detecting periapical pathosis on CBCT images.
Mackie [20]	- Study focuses on TMJ OA diagnosis using bone imaging biomarkers.	- Investigated the articular fossa radiomic biomarkers and condyle-to-fossa distance, noting differences in the condyle-to-fossa distance between control and TMJ OA patients utilizing a LightGBM (light gradient boosting machine) model.	- No statistically significant difference in articular fossa radiomic biomarkers between TMJ OA and control patients.	- Articular fossa imaging features may have a larger contribution in diagnosis.
Tajima [18]	- AI system detects cyst-like lesions in jaws on panoramic radiographs.	- Deep learning algorithm with transfer learning used for training data. - Development of a deep convolutional neural network (DCNN) for automatic detection.	- Results included detection of cyst-like radiolucent lesions on panoramic radiographs. - Identified lesions: radicular cysts, dentigerous cysts, odontogenic keratocysts, simple bone cysts, and ameloblastomas.	- AI system detected cyst-like lesions with high accuracy. - AI may contribute to diagnostic support in future clinical practice.
Fukuda [17]	- Compares three CNNs for mandibular third molar and canal relationship. - Evaluated time, storage, diagnostic performance, and consistency of CNNs.	- A DCNN was constructed using transfer learning techniques to automatically detect the lesions.	- Good or very good consistency values for all CNNs. - No significant differences in diagnostic performance among CNNs with smaller patches.	- Time and storage requirements depended on CNN depth and parameters. - Image patch size crucial for high diagnostic performance and consistency.
Ariji [16]	- Deep learning detects mandibular radiolucent lesions with high sensitivity.	- Utilized 210 training images with corresponding labels for model training on a deep learning GPU training system (DIGITS) using the DetectNet neural network.	- Sensitivity was 0.88 for both testing 1 and 2. - False-positive rate per image was 0.00 for testing 1. - False-positive rate per image was 0.04 for testing 2.	- Deep learning can achieve high sensitivity in radiolucent lesion detection in the mandible. - Dentigerous cysts showed best detection and classification sensitivity.
Shaheen [15]	- Develops AI system for tooth segmentation and classification on CBCT.	- Developed an artificial intelligence framework using a three-step segmentation approach, each step employing a 3D U-Net architecture.	- AI model outperformed ground truth with 0.56 ± 0.38 mm Hausdorff distance - AI was 1800 times faster than an expert in teeth segmentation	- 3D U-Net AI system was accurate and time-efficient for tooth segmentation. - AI system reduced clinical workload in dental diagnostics and treatment planning.
Cantu [14]	- Compares the performance of the neural network of the caries lesions of different radiographic extension on bitewings test dataset against seven independent evaluators.	- Utilized a convolutional neural network (U-Net) for analysis. - Stratified analysis based on lesion depth, categorizing into enamel lesions and dentin lesions.	- Neural network accuracy: 0.80, dentists’ mean accuracy: 0.71. - Neural network sensitivity: 0.75, dentists’ sensitivity: 0.36. - Dentists’ specificity: 0.91, neural network specificity: 0.83.	- Dentists under-detected lesions, while the network slightly over-detected.
Lee [13]	- Deep CNNs for dental caries detection and diagnosis on radiographs.	- Utilized a pre-trained GoogLeNet Inception v3 CNN for preprocessing and transfer learning	- Diagnostic accuracies for premolar, molar, and both models were provided. - Premolar model had the best area under the ROC curve.	- Deep CNN algorithm showed good performance in detecting dental caries.
Kuwada [12]	- Deep learning systems classify impacted supernumerary teeth in maxillary incisor region.	- Three different learning models were developed using AlexNet, VGG-16, and DetectNet.	- VGG-16 showed significantly lower values compared to DetectNet and AlexNet.	- DetectNet and AlexNet had potential for classifying impacted supernumerary teeth.
Yılmaz [11]	- Decision support system for classifying dental lesions using CBCT imaging.	- Utilized 50 CBCT images identified as periapical cysts and keratocystic odontogenic tumors, based on clinical, radiographic, and histopathologic features. Custom-developed software was utilized for segmentation.	- SVM classifier achieved 100% accuracy and F1 scores. - SVM showed 96.00% accuracy and 96.00% F1 scores.	- Periapical cyst and KCOT lesions can be classified with high accuracy. - The study contributed to computer-aided diagnosis of dental apical lesions.

**Table 3:** Advanced AI applications in oral surgery.

Authors	Summarized Abstract	Methods Used	Results	Conclusions
Tanikawa [32]	- AI systems predict facial morphology post orthognathic surgery and orthodontic treatment.	- Collected lateral cephalograms and 3-D facial images pre- and post-treatment to develop two AI systems (System S and System E) using landmark-based geometric morphometric methods combined with deep learning.	- Systems S and E had average errors of 0.94 mm and 0.69 mm. - Success rates for Systems S and E were 54% and 98%.	- Developed AI systems predict facial morphology after orthognathic surgery and orthodontic treatment. - AI systems are confirmed to be clinically acceptable for predicting facial morphology.
Jeong [34]	- CNNs judge soft tissue profiles for orthognathic surgery using facial photos.	- A comparative study with 822 subjects divided into two groups of 411 each - Front and side facial photographs were taken; the VGG19 CNN model was employed for analysis.	- CNNs achieved 89.3% accuracy in judging soft tissue profiles. - Precision, recall, and F1 scores were 0.912, 0.867, and 0.889.	- CNNs can accurately judge soft tissue profiles for orthognathic surgery. - Deep learning networks are valuable for screening in dental field.
Zhang [30]	- Artificial neural networks predict postoperative facial swelling after third molar extraction.	- Evaluated an artificial neural network’s accuracy in predicting postoperative facial swelling after mandibular third molar extraction. - Employed an improved conjugate gradient BP algorithm.	- Artificial neural network model predicted postoperative facial swelling with 98.00% accuracy.	- Improves conjugate gradient BP algorithm enhances prediction accuracy.
Kim [31]	- Investigated image patterns in cephalometric radiographs for orthognathic surgery diagnosis.	- Involved 960 patients split between those requiring orthognathic surgery (320) and non-surgical treatments (640). - Utilized CNN models ResNet-18, 34, 50, and 101.	- ResNet-18 had the best performance in screening with an AUC of 0.979. - Average success rates for ResNet models ranged from 91.13% to 93.80%.	- Study outlines characteristics needed for medical image-based decision-making models.
Choi [33]	- AI model determined mandibular third molar (M3) and inferior (alveolar nerve) IAN position in panoramic radiographs.	- Utilized 571 panoramic images to develop an AI model using ResNet-50 to determine the positional relationship between M3 and IAN.	- AI accuracy for true contact position was 72.32%, superior to OMFS. - AI accuracy for bucco-lingual position was 80.65%, surpassing OMFS specialists.	- AI model shows higher accuracy in bucco-lingual position determination. - AI can support clinicians in decision making for M3 treatment.

**Table 4:** Advanced AI applications in orthodontics.

Authors	Summarized Abstract	Methods Used	Results	Conclusions
Niño-Sandoval [35]	- Predicted mandibular morphology using automated learning techniques in Colombian patients.	- Automated learning techniques: artificial neural networks and support vector regression. - Support vector regression (SVR) and artificial neural networks (ANNs).	- Coefficients ranged from 0.84 to 0.99 with artificial neural networks. - Support vector regression achieved two coefficients above 0.7.	- Automated learning techniques predict mandibular morphology accurately. - Craniomaxillary variables can be used for facial reconstruction.
Panesar [36]	- AI improved precision and accuracy of cephalometric analyses significantly.	- Deep learning AI with and without human augmentation.	- AI improved precision and accuracy in cephalometric analyses significantly.	- AI/human augmentation method enhances less experienced dental professionals’ performance.
Alessandri-Bonetti [37]	- AI-assisted cephalometric analysis compared to manual software, showing reliability.	- Dahlberg equation for intra-and inter-operator reliability in cephalometric parameters.	- No significant difference in intra-and inter-operator measurements in cephalometric parameters. - Higher errors observed in posterior facial height and facial axis angle.	- Fully automated AI-assisted cephalometric software shows reliable and accurate measurements. - Digital advances cannot replace the orthodontist’s role in diagnosis.
Kochhar [38]	- Evaluated bone volume reliability in cleft lip and palate patients.	- Evaluation of bone volume using OsiriX software in CBCT scans. - Assessment of bone volume reliability by three specialists.	- Left-side clefts required more bone volume than the right side. - Age and gender did not correlate with bone volume needed.	- OsiriX software shows good reliability in bone volume measurements.
Çoban [39]	- Compared DM and AI cephalometric analysis in different skeletal malocclusions.	- Digital manual cephalometric analysis with Dolphin Imaging software (v. 11.5, California, USA). - AI-based cephalometric analysis using the WebCeph platform.	- Significant differences in most parameters between digital manual and AI methods.	- AI method needs further development for specific malocclusions. - Both AI and manual methods are suitable for orthodontic analysis.
Jiang [40]	- AI system for cephalometric analysis with automated landmark localization proposed.	- Collection of 9870 cephalograms from 20 medical institutions for training. - Development of a two-stage convolutional neural network for landmark localization.	- Average landmark prediction error was 0.94 ± 0.74 mm. - System achieved an average classification accuracy of 89.33%.	- Proposed AI system for cephalometric analysis improves diagnostic efficiency.
Li [41]	- ANN predicted orthodontic treatment plans with high accuracy and feasibility. - Study explores RBES, CBES, and ANN for orthodontic treatment planning.	- Multilayer perceptron artificial neural networks for orthodontic treatment planning. - Rule-based expert systems and case-based expert systems were utilized.	- Neural network accuracy: 94.0% for extraction–nonextraction prediction. - Extraction patterns accuracy: 84.2%. Anchorage patterns accuracy: 92.8%.	- Artificial neural networks aid in accurate orthodontic treatment planning. - MLPs show high accuracy in predicting extraction–nonextraction, extraction, and anchorage patterns.
Silva [42]	- CEFBOT (RadioMemory Ltd., Belo Horizonte, Brazil) AI software reliable for cephalometric landmark annotation and measurements.	- Cephalometric landmark annotation and measurements using AI-based software. - Duplicate measurements by human examiner and CEFBOT for reliability assessment.	- Frankfurt horizontal plane–true horizontal line angular measurement had the lowest reproducibility. - CEFBOT was unable to measure the distance from the glabella to the subnasale.	- No statistically significant difference between human examiner and CEFBOT measurements. - AI-based CEFBOT is comparable to human examiners in reproducibility.

**Table 5:** Advanced AI applications in pediatric dentistry.

Authors	Summarized Abstract	Methods Used	Results	Conclusions
You [48]	- AI model detected dental plaque on primary teeth with high accuracy.	- A pediatric dentist manually identified plaque on photos before and after applying a plaque-disclosing agent. Compared AI and manual diagnostic methods on an additional 102 intraoral photos.	- Mean intersection over union for detecting plaque was 0.726 ± 0.165. - AI model had higher MIoU compared to the dentist.	- AI model performs well in detecting dental plaque on primary teeth. - AI technology has the potential to enhance pediatric oral health.
Bilgir [47]	- AI system detected and numbers teeth on panoramic radiographs successfully.	- Developed AI (CranioCatch, Eşkişehir, Turkey) to detect and number teeth, tested on 249 panoramic radiographs.	- AI system successfully detected and numbered teeth on panoramic radiographs. - Estimated sensitivity, precision, and F-measure were 0.9559, 0.9652, 0.9606.	- AI can support clinicians, potentially replacing human observers in the future.
Duman [50]	- CNN-based AI model detected taurodontism in panoramic radiography effectively.	- Utilized 434 anonymized panoramic radiographs for developing automatic taurodont tooth segmentation models with a Pytorch-implemented U-Net.	- Sensitivity, precision, and F1-score values CNN system for taurodont tooth segmentation achieved results close to expert level in detecting taurodontism.	- CNN identifies taurodontism with results close to expert level.
Çalışkan [46]	- Deep learning for submerged primary tooth classification and detection.	- Employed Faster R-CNN architecture for detecting and classifying submerged molars. - Process involved defining molar boundaries on radiographs.	- Deep CNN showed high specificity in detecting submerged molars on radiographs. - AI approaches like deep CNN were promising for interpreting dental images.	- Further studies needed to enhance sensitivity for clinical application.
Ahn [45]	- Developed deep learning models for mesiodens classification in panoramic radiographs.	- Developed using SqueezeNet, ResNet-18, ResNet-101, and Inception-ResNet-V2.	- ResNet-101 and Inception-ResNet-V2 had accuracy over 90%. - SqueezeNet showed relatively inferior results in mesiodens classification.	- Deep learning models can aid in accurate and faster diagnosis.
Zhao [44]	- Developed AI tool for adenoid hypertrophy assessment in children.	- Developed an automated tool for adenoid hypertrophy assessment using a convolutional neural network, assessing regions defined by four key landmarks.	- High sensitivity, specificity, and accuracy for adenoid hypertrophy assessment. - Area under the receiver operating characteristic curve was 0.987.	- Automated system accurately assesses adenoid hypertrophy from lateral cephalograms.
Koopaie [49]	- Study compared cystatin S levels in early childhood caries patients. - Conducted a cross-sectional, case–control study.	- Collected unstimulated whole saliva samples using suction and measured cystatin S concentrations via ELISA. - Machine learning used to predict ECC based on cystatin S.	- Salivary cystatin S levels were significantly lower in early childhood caries. - Machine learning methods improved ECC prediction with cystatin S levels.	- Machine learning models enhance ECC prediction using cystatin S and demographics.

**Table 6:** Advanced AI applications in periodontology.

Authors	Summarized Abstract	Methods Used	Results	Conclusions
Chau [51]	- AI may be useful for providing automated visual plaque control advice based on intraoral photographs.	- Collection of intraoral photographs labeled as healthy, diseased, or questionable. - Analysis of accuracy in gingivitis detection using artificial intelligence system.	- AI system had sensitivity of 0.92 and specificity of 0.94. - Mean intersection over union of the system was 0.60.	- Potential for monitoring patients’ plaque control effectiveness using AI system.
Lin [52]	- Proposes automatic alveolar bone loss measurement system for periodontitis diagnosis.	- TSLS and ABLifBm for teeth contours and bone loss areas. - CEJ_LG method for CEJ, ALC, and APEX localization.	- More than half of bone loss measurements within 10% deviation - All bone loss measurements within 25% deviation from ground truth.	- The proposed system effectively estimates horizontal alveolar bone loss. - The system can aid in early and accurate diagnosis of bone loss.
Chang [53]	- Develops hybrid method for staging periodontitis using deep learning architecture.	- Hybrid framework of deep learning and conventional CAD processing. - Automatic detection and classification of periodontal bone loss.	- Mean absolute differences between periodontitis stages were insignificant. - Overall ICC value between developed method and radiologists’ diagnoses was 0.91.	- Hybrid framework shows high accuracy in automatic periodontitis diagnosis. - Automatic method has high reliability compared to radiologists’ diagnoses.
Thanathornwong [54]	- Deep learning detects periodontally compromised teeth in digital panoramic radiographs.	- Utilized a faster R-CNN for periodontally compromised teeth detection. - Model used a pretrained ResNet architecture for detection.	- Faster R-CNN detected periodontally compromised teeth with 0.81 precision. - Model excluded healthy teeth areas, showing a recall rate of 0.80.	- Application of a faster R-CNN reduces diagnostic effort and enables automated screening.
Lee [55]	- Develops deep CNN algorithm for diagnosing and predicting periodontally compromised teeth.	- Combined pretrained deep CNN architecture with self-trained network. - Used periapical radiographic images for optimal CNN algorithm.	- Deep CNN algorithm had 81.0% diagnostic accuracy for premolars. - Predicted extraction accuracy was 82.8% for premolars.	- Deep CNN algorithm useful for diagnosing and predicting periodontally compromised teeth.
Alotaibi [56]	- Develops CNN algorithm for bone loss detection in dental radiographs.	- Utilized pre-trained deep CNN architecture and self-trained network. - CNN (VGG-16) used for detecting alveolar bone loss in radiographs.	- Deep CNN algorithm detected alveolar bone loss with 73% accuracy. - Model classified severity of bone loss with 59% accuracy.	- Deep CNN algorithm useful in detecting alveolar bone loss. - Machines can perform better in image diagnosis with deep learning.

**Table 7:** Advanced AI applications in prosthodontics.

Authors	Summarized Abstract	Methods Used	Results	Conclusions
Lee [57]	- Deep CNN algorithm accurately identified and classifies dental implant systems.	- Image preprocessing and transfer learning techniques were applied. - Utilized fine-tuned and pre-trained deep CNN architecture (GoogLeNet Inception-v3).	- Inception-v3 architecture demonstrated the best performance for classification tasks. - Straumann BLT implant system had the highest accuracy among implant types.	- Deep CNN architecture effective for dental implant system identification and classification.
Bayrakdar [58]	- AI system evaluated for dental implant planning in CBCT images.	- Manual assessment with InvivoDental 6.0 for bone height and thickness. - Deep convolutional neural network (Diagnocat, Inc., San Francisco, USA) for evaluations.	- AI system showed no significant differences in bone height measurements.	- AI systems aid in implant planning, supporting physicians in practice. - AI demonstrates high detection percentages for canals, sinuses, and missing teeth.
Lerner [59]	- AI used to fabricate implant-supported zirconia crowns with high success. - Study protocol included CAD design, milling, sintering, and clinical application.	- Intraoral scan of implant position, CAD design of abutment, milling of zirconia abutment, clinical application of hybrid abutment.	- Quality of fabrication of hybrid abutments had a mean deviation of 44 μm. - Three-year cumulative survival and success rates for MZCs were 99.0% and 91.3%.	- MZCs show excellent marginal adaptation, interproximal, and occlusal contacts.
Takahashi [60]	- Deep learning identified dental implants from radiographic images effectively. - Object detection algorithm used to identify six implant systems accurately.	- Deep learning with Yolov3 algorithm for implant system identification. - Utilized TensorFlow and Keras deep learning libraries for implementation.	- True positive ratio and average precision of implant systems evaluated.	- Implants can be identified from panoramic radiographic images using deep learning. - Identification system could assist dentists and patients with implant related problems.
Yamaguchi [61]	- AI predicted debonding probability of CAD/CAM CR crowns from 2D images.	- Deep learning with a CNN method to predict debonding probability. - Utilized STL models of die, adjacent teeth, and antagonist.	- Mean calculation time for test images was 2 ms/step. - Area under the curve (AUC) value was 0.998.	- Deep learning with CNN predicts CAD/CAM CR crown debonding probability accurately.

## Data Availability

Data sharing is not applicable.
